# CASE REPORT Case Report and Review of the Literature: Deep Inferior Epigastric Perforator Flap for Breast Reconstruction After Abdominal Recontouring

**Published:** 2012-12-03

**Authors:** Jonathan Bank, Lucio A. Pavone, Iris A. Seitz, Michelle C. Roughton, Loren S. Schechter

**Affiliations:** ^a^Section of Plastic and Reconstructive Surgery, Department of Surgery, University of Chicago Medical Center, Chicago, IL; ^b^University Plastic Surgery, LLC, Morton Grove, IL

## Abstract

**Objective:** The report herein presents a case of a 49-year-old woman with left breast cancer who presented seeking immediate autologous reconstruction. Surgical history included an abdominal hysterectomy and an abdominal contouring procedure. This is a first description of a deep inferior epigastric perforator flap after abdominal wall manipulation of this magnitude. **Methods:** Computed tomographic angiography identified patent medial row perforators. Doppler confirmed the location of the perforators. The flap was designed with the inferior incision at the previous lower abdominal scar. Laser-assisted indocyanine green imaging confirmed adequate flap perfusion on the basis of a single left deep inferior epigastric perforator. **Results:** The flap was harvested on one perforator and anastomosed to the internal mammary system. The postoperative course was complicated by venous anastomosis kinking, requiring revision, but otherwise unremarkable. **Conclusion:** Computed tomographic angiography confirmed presence of perforators, communication with the deep inferior epigastric system, and location acceptable for flap design. Laser-assisted indocyanine green angiography facilitated perforator selection and provided intraoperative assessment of flap perfusion. Utilization of these modalities allowed safe completion of an operation considered contraindicated by conventional algorithms and highlights their role in complex perforator flap reconstruction.

Deep inferior epigastric perforator (DIEP) flaps have been reported in patients with vertical and short transverse abdominal scars after intra-abdominal surgery.^1^ A recently published retrospective study by Mahajan et al^2^ demonstrated the safety of performing perforator-based breast reconstruction in a cohort of patients with Pfannenstiel incisions. Even after more extensive manipulation of the abdominal wall, such as abdominoplasty and liposuction, authors have reported a short series of breast reconstruction with transverse rectus abdominis muscle free flaps.^3^

Clearly, as the body of experience grows, microsurgeons are becoming more aggressive in their surgical efforts. We seek to do so while maintaining patient safety and increasing procedural reliability. The objective of this report was to propose that advanced imaging modalities might permit perforator-based reconstruction in patients previously thought to be ineligible because of prior surgery.

## CASE DESCRIPTION

A 49-year-old woman with left breast cancer (ductal carcinoma in situ as well as invasive ductal carcinoma pT1N0M0) was seen in consultation seeking immediate autologous breast reconstruction. Review of her surgical history revealed an abdominal contouring procedure performed by another surgeon approximately 6 years prior to presentation. This surgery included resection of skin and fat of the lower abdomen and elevation of an adipocutaneous flap above level of umbilicus. A hysterectomy was performed at the time by a vertical fascial incision. The umbilicus was translocated. Of note, the rectus fascia was not plicated.

**Figure 1:**
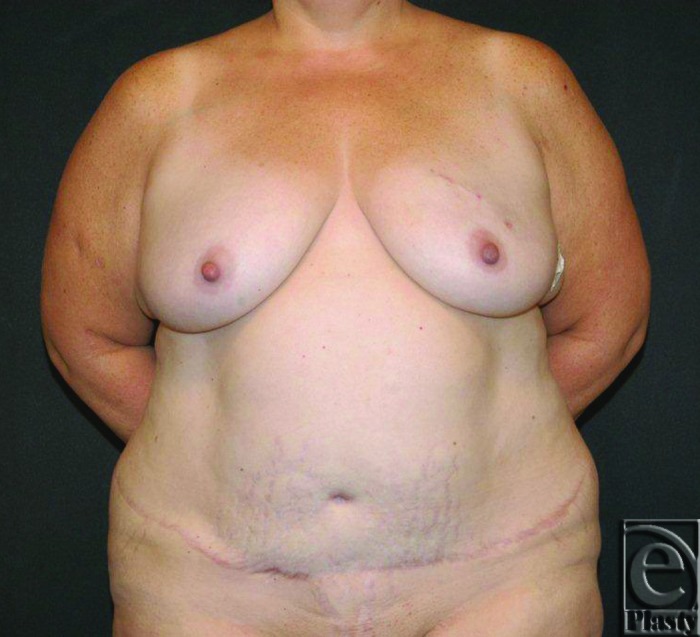
Preoperative photograph showing a long transverse abdominal scar.

Physical examination revealed moderate sized breasts with grade 2 ptosis. Abdominal scars included a low transverse incision from hip to hip and a periumbilical scar. Adequate abdominal soft tissue for bilateral reconstruction was present, whereas medial thigh and gluteal donor sites were deemed insufficient (Fig 1).

## METHODS

**Figure 2:**
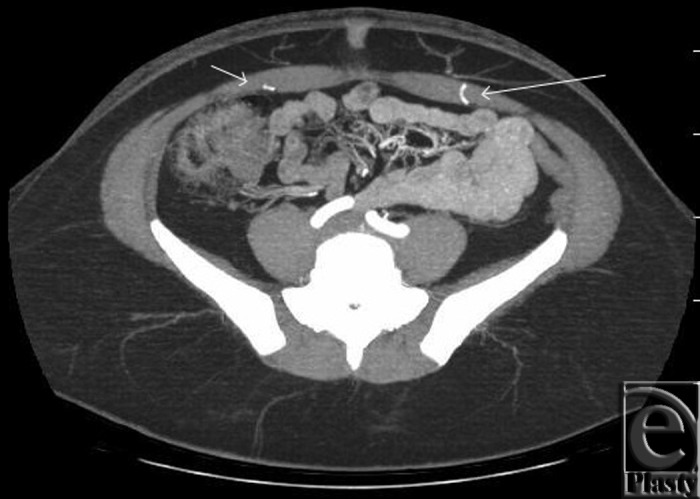
Right (short arrow) and left (long arrow) deep inferior epigastric perforators on preoperative computed tomographic angiography.

Preoperative evaluation included a computed tomographic angiography. Bilateral deep inferior epigastric artery perforators were identified. These perforators were located approximately 4 cm caudal and lateral to the umbilicus on either side. The right-sided perforator measured 1 mm in diameter, and the left-sided perforator measured 2 mm. Both perforators were traceable to their origin from the deep inferior epigastric artery (Fig 2).

The mastectomy specimen weighed 942 g. The flap was centered on the radiographically identified perforators. The lower portion of the abdominal incision incorporated the previous transverse scar, and the flap dissection proceeded cephalad, allowing identification of the perforators on each side. At this point, given the prior surgery and stretching of the upper abdominal wall soft tissue, in addition to standard clinical signs, laser-assisted indocyanine green (ICG) fluorescence imaging was used to assess flap perfusion. The right-sided perforators were temporarily clamped, ICG was injected, and the SPY Elite system (LifeCell Corporation, Branchburg, NJ, USA) was used to obtain the images.

**Figure 3:**
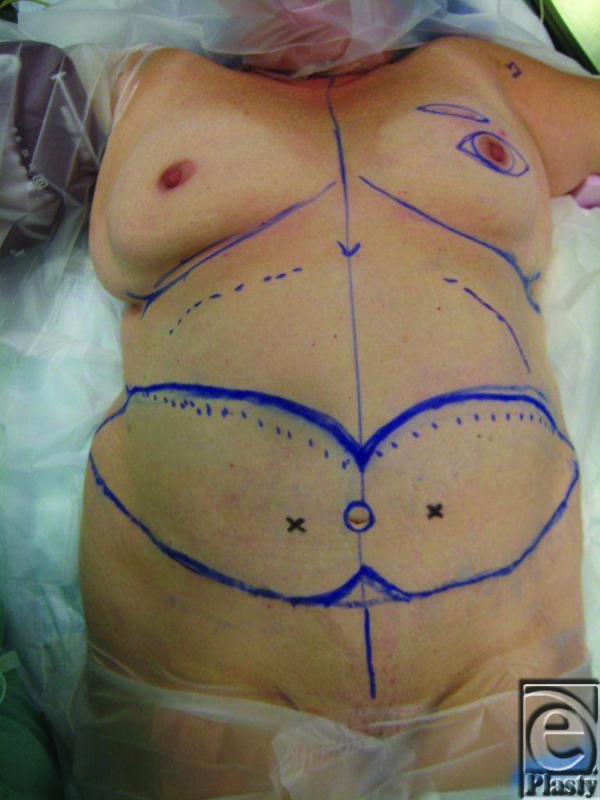
Initial flap design with markings of the right and left perforators found on Doppler auscultation.

**Figure 4:**
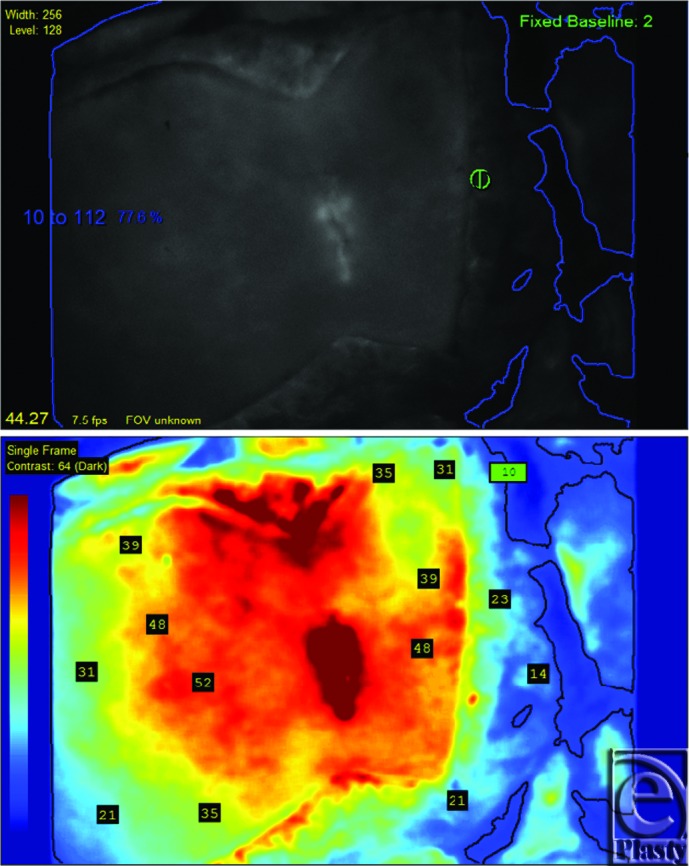
Intraoperative laser-assisted indocyanine green angiography showing absolute fluorescence (top) and relative fluorescence (bottom), based on a single left deep inferior epigastric artery perforator. Note that the orientation of the image is reversed as the SPY Elite probe was aimed in a cephalad-cuadad direction.

The abdominal flap demonstrated perfusion in zones 1, 2, and 3, based on the single left-sided medial row deep inferior epigastric artery perforator (Fig 3). The flap was harvested on the basis of the single, left-sided perforator. Zone 4 (the right lateral portion of the flap) was discarded. Anastomoses were performed between the left deep inferior epigastric artery and vein and the left internal mammary artery and vein in an end-to-end fashion. Repeat laser-assisted ICG imaging revealed good flap perfusion after inset (Fig 4).

## RESULTS

**Figure 5:**
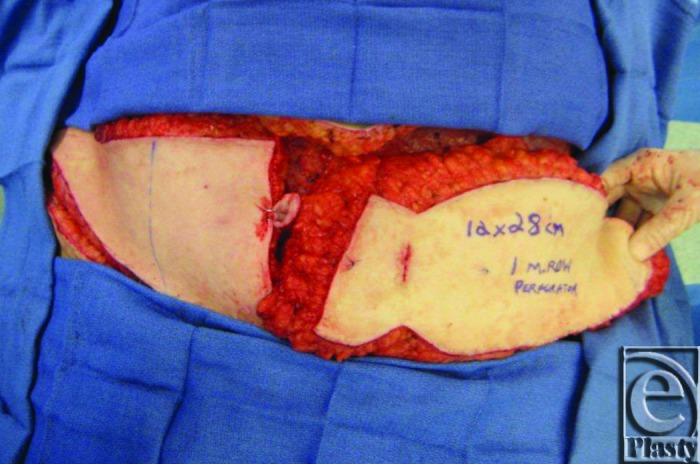
Final flap in situ based on a single left deep inferior epigastric artery perforator.

**Figure 6:**
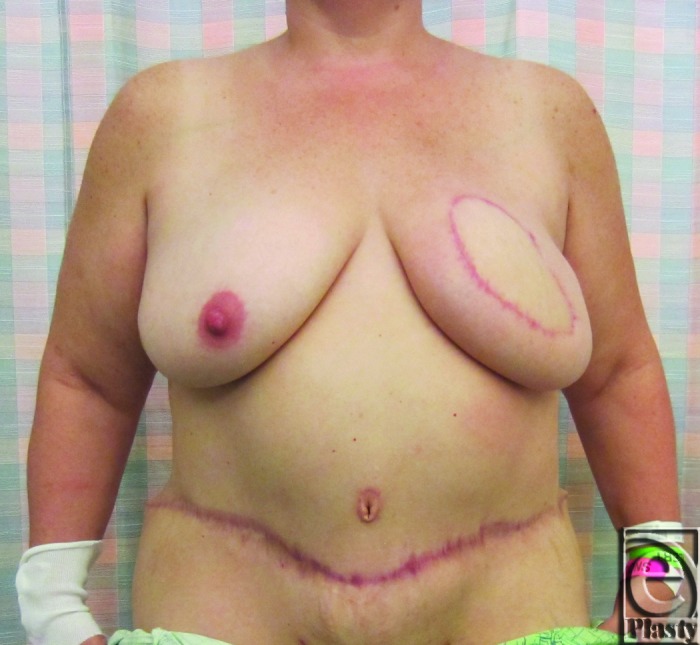
Result 4 months postoperatively, prior to scar revisions and symmetry procedures.

The early postoperative course was complicated by venous congestion 2 hours after surgery. Exploration revealed a kink at the coupled venous anastomosis. The venous anastomosis was revised, and the remainder of the patient's course was unremarkable (Figs 5 and 6).

## DISCUSSION

Cases of DIEP flaps in patients with vertical abdominal scars have been reported in the literature.^4^^,^^5^ However, long, transverse incisions are generally considered to be a contraindication for this procedure.^1^

In 2010, Jandali et al^3^ retrospectively reported a series of abdominally based flaps for breast reconstruction after cosmetic abdominal surgery. This series included 3 cases of free transverse rectus abdominis myocutaneous flap breast reconstruction after abdominoplasty and 3 cases of free transverse rectus abdominis myocutaneous or DIEP flaps after abdominal liposuction. In addition, De Frene et al^6^ reported DIEP flap reconstruction after liposuction. In this series, color Doppler was used to assess perforator patency. In both series, the authors suggested that vascular ingrowth after the initial cosmetic procedure may have enabled the subsequent reconstruction. While this may seem feasible for a transverse rectus abdominis myocutaneous flap reconstruction based on multiple medial and lateral row perforators, this seems less likely for a DIEP flap.

In April 2012, Mahajan et al^2^ published a retrospective cohort study examining the impact of a Pfannenstiel incision on surgical anatomy and outcomes in DIEP breast reconstruction. The authors found that the presence of a Pfannenstiel incision is associated with a significant increase in the dimension of the remaining perforators (in a matched control cohort), favoring subsequent harvesting of a DIEP flap. They suggest a mechanism of “ischemic preconditioning,” although the study was not designed with the intent of establishing causality of perforator size change after Pfannenstiel incisions. Outcomes were similar with regard to flap loss, fat necrosis, wound healing, and abdominal wall laxity, providing further edification that DIEP flaps are feasible and safe after low transverse scar abdominal surgery.

The use of computed tomographic angiography to guide surgical planning for perforator-based reconstructing has been well described.^7^ Multiple authors have found that the computed tomographic angiography helps in planning surgery, selecting the optimal flap, and reducing operating time. In our practice, we use this method in most cases, particularly when the anatomy may be altered because of previous surgery of an unknown extent.

The introduction of laser-assisted ICG angiography^8^ may aid intraoperative clinical decision making.^9^ It can assist in isolating perforators on the abdominal wall preoperatively, help determine which perforator system provides optimal tissue perfusion, assess patency of anastomoses, and discern areas of the flap that should be discarded upon harvest or after inset. In essence, it provides a more physiological evaluation of vasculature found to be anatomically present on computed tomographic angiography and on surgical exploration. In tissues that have been previously surgically manipulated, vessels encountered may not be as reliable as vessels in virgin tissue for the purpose of flap survival. We find that laser-assisted ICG angiography potentially decreases the uncertainty of perfusion patterns in these cases, thereby increasing surgeon's confidence in flap design and, ultimately, raising surgical reliability.

Conventional “abdominoplasty” may have different interpretations and executions by different surgeons, with varying degrees of cephalad undermining and perforator preservation. This variability and resultant perceived flap unreliability has rendered perforator-based reconstructions after extensive abdominal wall procedures “contraindicated.” However, as perforator-sparing techniques are progressively used and engrained across various disciplines of plastic surgery, specifically in abdominal wall procedures, should the need arise, the opportunity to use these perforators in free tissue reconstruction need not be discounted. We suspect that, in this case, the perforators superior to the umbilicus were not injured during the patient's initial procedure despite noted dissection above this level. The case presented illustrates the utility of advanced imaging modalities in delineating surgically altered vascular anatomy and physiology.

The combination of preoperative computed tomographic angiography for anatomic assessment and intraoperative laser-assisted ICG angiography for perfusion assessment may allow extension of perforator-based reconstruction in scenarios previously considered contraindicated.^10^ To date, this is the first case of a DIEP flap reported after extensive abdominal wall recontouring. We encourage the judicious use of these imaging modalities in relevant scenarios.
